# Hepatitis C and not Hepatitis B virus is a risk factor for anti-tuberculosis drug induced liver injury

**DOI:** 10.1186/s12879-016-1344-2

**Published:** 2016-02-01

**Authors:** Wan Soo Kim, Sang Soo Lee, Chang Min Lee, Hong Jun Kim, Chang Yoon Ha, Hyun Jin Kim, Tae Hyo Kim, Woon Tae Jung, Ok Jae Lee, Jeong Woo Hong, Hyun Seon You, Hyun Chin Cho

**Affiliations:** 1 Department of Internal Medicine, Gyeongsang National University Hospital, Gyeongsang National University School of Medicine, Gangnam-ro 79, Jinju, Gyeognam 660-702 South Korea; 2 Department of Internal Medicine, Samsung Changwon Hospital, Sungkyunkwan University School of Medicine, Palyong-ro, 158, MasanHoiwon-gu, Chang-Won, Republic of Korea

**Keywords:** Tuberculosis, Treatment, Hepatitis B virus, Hepatitis C virus, Drug induced liver injury

## Abstract

**Background:**

The risk of anti-tuberculosis (TB) drug-induced liver injury (DILI) in patients with chronic viral hepatitis (CVH) is not clear. The aim of this study was to investigate incidence and risk factors associated with TB DILI in CVH and non-CVH patients.

**Methods:**

Retrospectively, a total of 128 CVH patients who received anti-TB medication from January 2005 to February 2014 were reviewed. Among these, 83 patients had hepatitis B virus (HBV), 41 patients had hepatitis C virus (HCV) and 4 patients were dual hepatitis B and hepatitis C virus co-infected (HBV + HCV) with 251 non-CVH patients who received anti-TB medication selected as the controls. There were no human immunodeficiency virus co-infected patients. Risk factors for DILI were analyzed using cox regression analysis.

**Results:**

The incidence of DILI was significantly higher in the HCV group (13/41 [31.7 %], *p* < 0.001) and HBV + HCV groups (3/4 [75.0 %], *p* = 0.002) compared to the control group (25/251 [10.0 %]). The incidence of transient liver function impairment in the hepatitis B virus group was higher than in the control group (18/83 [21.7 %] vs. 27/251 [10.8 %] *p* = 0.010), but not in DILI (11/83 [13.3 %] vs. 25/251 [10.0 %], *p* = 0.400). In total patients, HCV, HBV + HCV co-infection, older age, and baseline liver function abnormality were independent factors of DILI.

**Conclusions:**

It is recommended to carefully monitor for DILI in patients with HCV or HBV/HCV co-infection, older age, and baseline liver function abnormality.

## Background

Standard anti-tuberculosis (TB) drugs, including isoniazid (INH), rifampin (RFP), ethambutol (EMB), and pyrazinamide (PZA) are highly effective in treating TB. However, drug induced liver injury (DILI) associated with anti-TB treatment is the most important adverse event, which results in a low treatment success rate [1]. The incidence of DILI during standard anti-TB treatment ranges from 2 % to 28 % [2–6]. Once DILI occurs, all anti-TB drugs should be withheld until a complete resolution of the hepatotoxicity is accomplished [7]. Advanced age, female gender, alcohol abuse, malnutrition, and underlying chronic liver disease have been reported to be significant risk factors for DILI during anti-TB treatment [8–10]. However, it remains unclear whether the incidence of DILI increases during anti-TB treatment in patients with chronic viral hepatitis (CVH). In a previous Hong Kong study [11], persistent liver dysfunction was shown to be more common in hepatitis B virus (HBV) infected patients. In addition, several recent studies have suggested that HCV infections are also a significant risk factor for incident DILI during anti-TB treatment [12–15]. However, in still other studies, the incidence of transient liver dysfunction during anti-TB treatment was found to be higher in HBV or HCV infected patients than in patients without viral hepatitis, but the incidence of DILI was not different between CVH and non-CVH patients [16–20].

In Korea, both TB and CVH are major public health concerns. The number of new onset TB patients is about 40,000 per year in Korea and is continuing to increase [21]. The prevalence of HBV in Korean population is 3.7 % [22], and 0.78 % of the Korean population has an HCV infection [23]. The aim of present study was investigate the incidence and risk factors of DILI in patients with CVH and to compare them to patients without CVH.

## Materials and Methods

### Study populations

We retrospectively enrolled a total of 128 patients with CVH who were consecutively diagnosed with pulmonary or extrapulmonary TB and receiving standard anti-TB drugs at the Gyeongsang National University Hospital, a tertiary hospital in Korea, from January 2005 to February 2014. Among these, 83 patients were positive for HBV surface antigen (HBsAg) and negative for the HCV antibody (HBV group), 41 were negative for HBsAg and positive for the HCV antibody (HCV group), and 4 were positive for both HBsAg and HCV antibody (HBV + HCV group). The following data were collected from electronic medical records by computer-assisted chart review; age, sex, alcohol ingestion, body mass index (BMI), previous TB history, TB infection site (lung or other), date of anti-TB drugs prescription, regimen of anti-TB drugs, HBsAg, HCV antibodies, HBV DNA, HCV RNA, serial liver function test once in 2 weeks, including aspartate aminotransferase (AST), alanine aminotransferase (ALT), and total bilirubin, symptoms of hepatitis including anorexia, nausea, vomiting, generalized weakness, abdominal discomfort, and jaundice, and comorbidities including liver cirrhosis, chronic kidney disease, diabetes mellitus, hepatocecullar carcinoma, and other malignancies.

For the purposes of comparison, 251 consecutively patients diagnosed with pulmonary or extrapulmonary TB with negative HBsAg and HCV antibody from January 2013 to February 2014 were enrolled and their respective electronic medical records were analyzed as the control group. If a patient had liver function impairment, the time until onset was measured in days. The mean onset time and standard deviation were calculated. The incidence of DILI was investigated according to patients with and without viral hepatitis. Follow up duration was measured by 12 months in cases without hepatotoxicity. Patients with initial AST or ALT over 2 times the upper limit of normal, initial total bilirubin over 1.5 times the upper limit of normal, less than 3 months of treatment duration, and liver function abnormality caused by a source other than anti-TB drugs were excluded. There were no HIV-infected patients among the enrolled patients. The present study was approved by the Institutional Review Board of the Gyeongsang National University Hospital.

### Definitions

The liver function impairment was classified as transient liver function impairment (TLI) and DILI. The definition of TLI and DILI was based on previous studies and literature [1, 16–18, 24]. TLI was defined as AST/ALT levels above the upper limit of normal (40 IU/L), but were still less than three times the upper limit of normal (120 IU/L) and resolved spontaneously, despite continued medication. DILI was defined as liver transaminase levels were exceeded 120 IU/L with symptoms of hepatitis or exceeded 200 IU/L without symptoms. If AST/ALT levels were under 200 IU/L, the case was defined as mild DILI. AST/ALT levels of 200 to 500 IU/L indicated moderate DILI, and AST/ALT levels exceeding 500 IU/L were considered to indicate severe DILI. A baseline liver function test (LFT) abnormality was defined as AST or ALT over 40 IU/L. Alcohol consumption was defined as an intake of more than 30 g/day of alcohol for males and 20 g/day for females [25].

### Anti-tuberculosis treatment

In South Korea, treatment of TB is based on the National Tuberculosis Guidelines [26]. They are nearly identical to American and World Health Organization (WHO) guidelines [1, 27]. The recommended treatment is a 6 month regimen consisting of 2 months of INH, RFP, EMB, and PZA, followed by 4 months of INH and RFP. Most patients received daily drugs including INH (300 mg), RFP (450 ~ 600 mg), EMB (800 ~ 1200 mg), and PZA (1,500 mg). After patients develop DILI, additional modes of anti-TB management are used. Patients with DILI are managed in one of two ways based on the physician’s judgment and treatment guidelines [26]: (1) cease the anti-TB treatment and restart treatment when liver transaminase levels fall to less than two times the upper limit of normal, or (2) cease the anti-TB treatment and restart the alternative treatment with non-hepatotoxic drugs or cease the anti-TB treatment and do not restart treatment.

### Statistical analysis

SPSS (version 21.0; IBM SPSS statistics) was used for statistical analysis. Continuous variables were compared using the Mann–Whitney U tests. Categorical variables were compared using Fisher’s exact test and the chi-square test. The cumulative incidences of the control, HBV, HCV, and HBV + HCV groups were analyzed using Kaplan-Meier curve and comparison of the differences of incidence was performed using the log-rank test. The association between DILI and the presence of HBV, HCV, CVH, or total patients was evaluated by Cox regression analysis. The risk was expressed by calculating the hazards ratio (HR) and 95 % confidential interval (CI). P-values under 0.05 were considered statistically significant.

## Results

### Patient characteristics

A total of 379 patients were enrolled in this study. Baseline characteristics of each group are presented in Table 1. There were no significant differences in sex, other malignancies, alcohol ingestion, extrapulmonary TB, BMI, or chronic kidney disease between groups. The control group was significant older than the HBV group. The rate of use of PZA was lower in the HBV group. Incidences of hepatocellular carcinoma and liver cirrhosis were higher in the CVH patients. Baseline LFT abnormalities were more common in CVH patients.

**Table 1:** Baseline characteristics of all patients and chronic viral hepatitis patients

	Control, non-CVH (*n*=251)	CVH		
		HBV (*n*=83)	HCV (*n*=41)	HBV+HCV (*n*=4)
Age, years	59.3±17.3	50.0±15.1^a^	59.9±14.9	54.3±9.5
Sex, female	155 (61.8)	61 (73.5)	27 (65.9)	4 (100)
Use of PZA	235 (93.6)	68 (81.9) ^a^	36 (87.8)	3 (75.0)
DM	49 (19.5)	18 (21.7)	17 (41.5) ^a^	2 (50.0)
HCC	1 (0.4)	7 (8.4) ^a^	2 (4.9)	0 (0)
Other malignancy	42 (16.7)	8 (9.8)	2 (4.9)	0 (0)
Alcohol ingestion^b^	42 (16.7)	9 (10.8)	7 (17.1)	1 (25)
Extrapulomary TB	72 (28.7)	24 (28.9)	8 (19.5)	0 (0)
Past TB history	30 (12.0)	19 (22.9) ^a^	7 (17.1)	2 (50)
BMI >25kg/m^2^	37 (16.5)	8 (10.3)	6 (15.4)	0 (0)
Liver cirrhosis	3 (1.2)	21 (25.3) ^a^	4 (9.8) ^a^	2 (50) ^a^
CKD	13 (5.2)	3 (3.6)	2 (4.9)	0 (0)
Baseline LFT abnormality^c^	27 (10.8)	21 (25.3) ^a^	15 (36.6) ^a^	2 (50)

Data are presented as mean ± standard deviation for continuous data and number (%) for categorical data. HBV: hepatitis B virus, HCV: hepatitis C virus, BMI: body mass index, LFT: liver function test, DM: diabetes mellitus, HCC: Hepatocellular carcinoma, TB: tuberculosis, CKD: Chronic kidney disease, PZA: pyrazinamide

^a^ *p*-value < 0.05 compared to control group using Fisher’s exact test or Mann-Whitney U test.

^b^Alcohol ingestion defined as consuming 30 g/day or more for males and 20 g/day or more for females.

^c^Baseline liver function abnormality was defined as aspartate aminotransferase or alanine aminotransferase over 40 IU/L.

### Incidence of hepatotoxicity

**Fig. 1:**
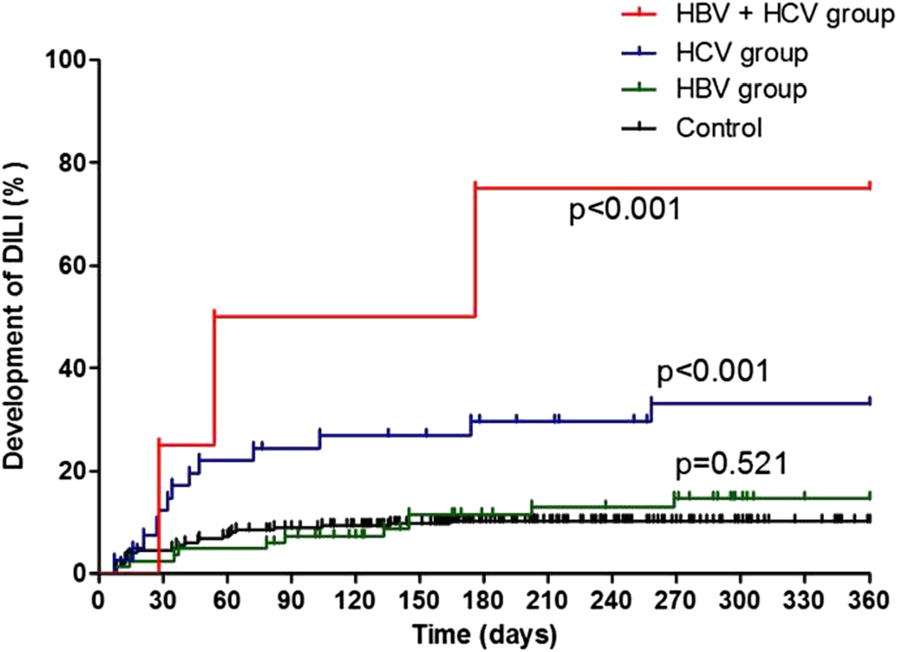
Kaplan-Meier curve for occurrence of drug-induced liver injury. The cumulative incidences of drug-induced liver injury in the hepatitis C virus (p = 0.001) and hepatitis B virus + hepatitis C virus groups (p = 0.001) were significantly higher than that of the control group, but not in the hepatitis B virus group (p = 0.521). P-value compared with control group, DILI, drug-induced liver injury; HBV, hepatitis B virus; HCV, hepatitis C virus

In total patients who received anti-TB treatment, DILI occurred in 13.7 % (52/379). The incidence of total hepatotoxicity (TLI + DILI) was higher in the HBV (34.9 %, *p* = 0.008), HCV (53.6 %, *p* = 0.001), and HBV + HCV groups (100 %, *p* = 0.002) than in the control group (20.7 %). In Table 2, it was shown that the HBV (21.7 %, *p* = 0.010) and HCV groups (22.0 %, *p* = 0.040) developed higher TLI than the control group (10.8 %). The incidence of DILI was significantly higher in the HCV (31.7 %, p < 0.001) and HBV + HCV groups (75.0 %, p = 0.002) compared to the control group (10.0 %), but not for the HBV group (13.3 %, p = 0.400). There were no significant differences in the frequencies of severe DILI in patients in the HBV, HCV, HBV + HCV, and control groups. In Kaplan-Meier analysis for the development of DILI, the incidence of DILI in the HCV (p = 0.001) and HBV + HCV groups (p = 0.001) were significantly higher than that of the control group, but not for the HBV group (p = 0.521) (Fig. 1).

**Table 2:** Incidence of transient liver function impairment (TLI) and drug-induced liver injury (DILI) during anti-tuberculous treatment in different patient groups

	Control (*n*=251)	HBV (*n*=83)	*P-*value*	HCV (*n*=41)	*P*-value†	HBV+HCV (*n*=4)	*P-*value‡
TLI	27 (10.8)	18 (21.7)	0.010	9 (22.0)	0.040	1 (25.0)	0.310
DILI	25 (10.0)	11 (13.3)	0.400	13 (31.7)	<0.001	3 (75.0)	0.002
Mild	12 (4.8)	2( 2.4)		7 (17.1)		2 (50.0)	
Moderate	8 (3.2)	6 (7.2)		5 (12.2)		1 (25.0)	
Severe	5 (2.0)	3 (3.6)		1 (2.4)		0 (0)	

HBV: hepatitis B virus, HCV: hepatitis C virus

*P-value: Control vs. HBV group

†*P*-value: Control vs. HCV group

‡*P*-value: Control vs. HBV+HCV group

### Risk factors associated with drug-induced liver injury

In Table 3, we analyzed the risk factors of DILI in the subject population with anti-TB treatment by Cox regression analysis. In univariate analysis, HCV infection, HBV + HCV co-infection, age, HCC, and baseline LFT abnormality were significant factors for DILI. In multivariate analysis, HCV infection (HR = 2.623, 95 % CI: 1.289-5.338, *p* = 0.008), HBV + HCV co-infection (HR = 9.401, 95 % CI = 2.691-32.841, *p* < 0.001), age (HR = 1.034, 95 % CI = 1.013-1.054, *p* = 0.001), and baseline liver function abnormality (HR = 2.331, 95 % CI = 1.238-4.390, *p* = 0.009) were independent risk factors for DILI.

**Table 3:** Cox regression analysis of risk factors for drug-induced liver injury in all patients

	Univariate analysis		Multivariate analysis	
	HR (95 % CI)	*P*-value	HR (95 % CI)	*P*-value
Control	Reference			
HBV	1.291 (0.635-2.624)	0.481	1.321 (0.612-2.850)	0.479
HCV	3.425 (1.752-6.697)	<0.001	2.623 (1.289-5.338)	0.008
HBV+HCV	9.273 (2.793-30.788)	<0.001	9.401 (2.691-32.841)	<0.001
Age, per year	1.025 (1.007-1.043)	0.005	1.034 (1.013-1.054)	0.001
HCC	3.361 (1.212-9.322)	0.020	1.790 0.598-5.356)	0.298
Baseline LFT Abnormality^a^	2.702 (1.526-4.784)	0.001	2.331 (1.238-4.390)	0.009

HBV: hepatitis B virus, HCV: hepatitis C virus, HCC: Hepatocellular carcinoma, HR: hazards ratio, CI: confidential interval, BMI: body mass index (kg/m^2^), LFT: liver function test

^a^Baseline LFT Abnormality: aspartate aminotransferase > 40IU/L or alanine aminotransferase > 40IU/L

We performed subgroup analysis to identify risk factors in each viral hepatitis group. In CVH patients, age (HR = 1.039, 95 % CI = 1.009-1.070, p = 0.011) and baseline LFT abnormality (HR = 4.601, 95 % CI = 2.017-10.496, *p* ≤ 0.001) were independent risk factors for the development of DILI (Table 4). In the HBV group, baseline LFT abnormality (HR = 8.643, 95 % CI = 1.598-46.748, *p* = 0.012), diabetes (HR = 8.819, 95 % CI = 1.228-63.352, *p* = 0.030), and HBV DNA ≥ 2000 IU/ml (HR = 5.140, 95 % CI = 1.150-22.978, *p* = 0.032) were predictors for the development of DILI. In the HCV group, age (HR = 1.091, 95 % CI = 1.014-1.173, *p* = 0.019) and baseline LFT abnormality (HR = 14.889, 95 % CI = 1.709-129.748, *p* = 0.014) were significant risk factors of DILI, but detection of HCV RNA was not a significant risk factor. High HBV DNA level (≥2,000 IU/ml) was an independent risk factor for DILI in the HBV group. HCV RNA PCR and HCV genotype were checked in only some of the patients (*n* = 29 and *n* = 14). However, the detection of HCV RNA was not significant factor for DILI in the HCV group.

**Table 4:** Cox regression analysis of risk factors for anti-tuberculosis drug-induced liver injury in chronic viral hepatitis, chronic hepatitis B, and chronic hepatitis C

	Multivariate analysis: hazard ratio (95 % confidential interval)		
Variables	CVH (*n*=128)	HBV (*n*=83)	HCV (*n*=41)
Age	1.039 (1.009-1.070)	0.990 (0.933-1.051)	1.091 (1.014-1.173)
Sex (female)		5.010 (0.518-48.478)	0.312 (0.059-1.639)
DM	2.064 (0.879-4.847)	8.819 (1.228-63.352)	
HCC	0.866 (0.253-2.966)		1.977 (0.309-12.632)
Liver cirrhosis			0.190 (0.026-1.362)
Baseline LFT abnormality^a^	4.601 (2.017-10.496)	8.643 (1.598-46.748)	14.889 (1.709-129.748)
HBV DNA ≥ 2000 IU/ml^b^		5.140 (1.150-22.978)	
HCV RNA detected^c^			0.361 (0.026~5.051)

DM: diabetes mellitus, HCC: Hepatocellular carcinoma, LFT: liver function test, HBV: hepatitis B virus, HCV: hepatitis C virus

^a^Baseline LFT Abnormality: aspartate aminotransferase > 40IU/L or alanine aminotransferase > 40IU/L

^b^ Among available data in 59 patients, 21 patients had high HBV DNA (≥2000 IU/ml)

^c^HCV RNA available (n=29), detected (n=24), not detected (n=5)

### Onset time of drug-induced hepatitis, modes of management and recovery time

**Fig. 2:**
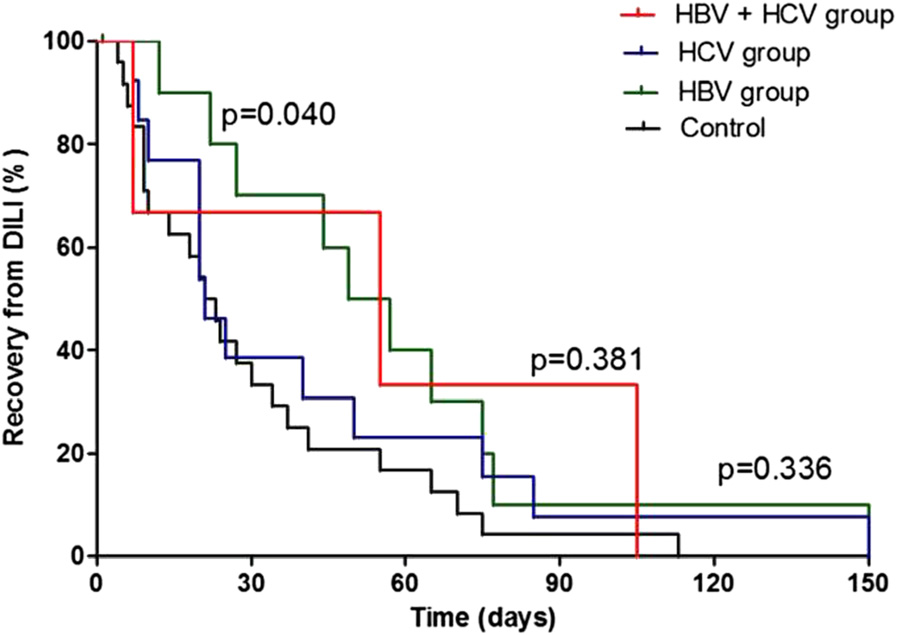
Kaplan-Meier curve for recovery from drug-induced liver injury. The recovery rate of drug-induced liver injury in hepatitis B virus (p = 0.040) was significantly higher in the early period than that of the control group, but not in the hepatitis C virus (p = 0.036) and hepatitis B virus + hepatitis C virus groups (p = 0.381). P-value compared with control group. DILI, drug-induced liver injury; HBV, hepatitis B virus; HCV, hepatitis C virus

There was no significant difference in mean onset time of DILI in patients with and without CVH (83.3 ± 77 days and 43.9 ± 41 days, p = 0.056). Among the 52 patients who developed DILI, 9/25 (36.0 %) of the control group and 3/27 (11.1 %) of CVH patients developed DILI within 2 weeks, which was statistically significant (*p* = 0.033, data not shown). There was no statistical significance between modes of management after the development of DILI between the control and CVH groups. Among 52 patients with DILI, 12/25 (48 %) and 11/27 (40.7 %) of patients successfully completed treatment with first-line drugs in the groups with and without CVH, respectively (*p* = 0.509, data not shown). The recovery time after the onset of DILI was longer in the HBV group than the control group, but not in the HCV and HBV + HCV co-infection group (Fig. 2).

## Discussion

In this study, DILI occurred in 13.7 % of total patients who received anti-TB treatment. It was found that HCV infection and HBV + HCV co-infection were independent risk factors for DILI. However, HBV infection was not a risk factor for DILI. The incidence of DILI was similar in patients with HBV infections without the presence of viral hepatitis. However, TLI was significantly higher in HBV-infected patients than in patients without viral hepatitis.

In previous studies [14, 16, 17], the incidence of DILI in HBV-infected patients was 7 % to 12 %. The incidence of DILI in HCV-infected patients was 9 % to 30 % [12–14, 16, 18]. In our study, the incidence of anti-TB DILI in the control group was 10.0 %. In CVH patients, the incidence of DILI was 13.3 %, 31.7 %, and 75.0 % in the HBV, HCV, and HBV + HCV groups, respectively. Compared to the control group, the incidence of DILI was significantly higher in the HCV and HBV + HCV groups, but not in the HBV group. However, when considering TLI in addition to DILI, the incidence was also statistically significant in the HBV group. There were 4 patients in our subject population that had both HBV and HCV infection. In this group, one patient showed TLI, while the remaining three developed DILI.

In some previous studies, it has been documented that HBV infections increase the risk for DILI. In Hong Kong, Wong et al. found that HBV infections were a significant risk factor for liver dysfunction. However, that study did not classified TLI and DILI, and only defined liver dysfunction as an increase in ALT levels to 1.5 times above the UNL [11]. If we applied that definition to our study, we would find a similar result: a significant incidence of any liver function impairment. In previous studies applied same definition of DILI [14, 16], the incidence of DILI in HBV patients were not significantly increased compared to normal control groups. In the HBV group, high HBV DNA level (≥2,000 IU/ml) is an independent risk factor for DILI (HR = 5.140, Table 4). In a previous study [28], it was recommended that using an antiviral agent to decrease the viral load to prevent the development of liver dysfunction. In the present study, among the 83 patients in HBV group, 16 patients were medicated with an antiviral agent, while 67 patients were not. The administration of an antiviral agent did not decrease the risk of DILI (data not shown).

In the HCV group, there was no statistical significance regarding the detection of HCV RNA, an HCV genotype. A previous study in Taiwan [24], found that a high initial HBV/HCV viral load was a significant risk factor for DILI. In another study, the detection of HCV viral load and HCV genotype were not significant predictors of DILI [12]. The mechanism accounting for the higher incidence of DILI in the HCV group than in the HBV group is not known yet. In a previous study, Wedemeyer et al. showed HCV core protein alters lipid metabolism, contributing to the development of hepatic steatosis. Hepatic steatosis may trigger hepatocyte apoptosis, which facilitates inflammation and fibrosis [29]. Other studies have explained that a high baseline viral load more frequently leads to the development of DILI [24]. A pro-inflammatory environment induced by actively replicating the hepatitis virus may alter the detoxication process and increase drug toxicity [30]. Although, we did not find any associations with BMI and hepatotoxcity, there are some studies indicating low BMI as a risk factor [31]. Further study is needed about the interaction of CVH and high incidence of DILI.

Previous studies have reported that DILI usually occurs within 2 months of the anti-TB treatment [32]. Liu et al. reported that TLI occurred later in CVH patients, but not for DILI [16]. In present study, DILI was more common in the CVH group than the control group after 2 weeks of treatment. In addition, there was no difference in the modes of treatment management of DILI between the control and CVH groups. In previous studies [16–18], the severity of DILI was not significantly different in the control and CVH groups. In another previous study [14], both the HBV and HCV groups had longer durations of AST/ALT elevation compared to the control group. In this study, the recovery time after the onset of DILI was longer in the HBV group than in the control group, but not in the HCV and HBV + HCV co-infection groups.

This study has some limitations. First, because of its retrospective nature, the study may provide inaccurate information about alcohol intake or herbal medication. Second, there was a lack of information about the hepatitis virus in CVH patients, HBV DNA and HCV RNA viral load, HCV genotype, etc. Third, this was a single-center study with a relatively small sample size, especially HBV + HCV co-infection group. Forth, there were so many baseline differences between the 2 main patient groups and the control group such that outcomes would be predicted to be different on this basis alone. Further prospective study with more patients is needed to gleam more detailed information about the hepatitis virus.

## Conclusion

HCV infection and HBV + HCV co-infection were associated with anti-TB treatment-induced hepatotoxicity. HBV infection was only associated with TLI. In the total patient population receiving anti-TB treatment, the independent risk factors for DILI were HCV with or without HBV infection, age, and baseline liver function abnormality. It is recommended to carefully monitor for DILI in patients with HCV or HBV + HCV co-infections, old age, and abnormal baseline LFTs.

## Abbreviations

TB: Tuberculosis
TLI: Transient liver function impairment, DIH, drug induced hepatitis
HBV: Hepatitis B virus
HCV: Hepatitis C virus
LFT: Liver function test
BMI: Body mass index
HR: Hazards ratio
CI: Confidence interval
CVH: Chronic viral hepatitis
